# Supplementary material for: Hemoglobin and Its Relationship with Fatigue in Long-COVID Patients Three to Six Months after SARS-CoV-2 Infection
Source: Biomedicines. 2024 Jun 1;12(6):1234. doi: 10.3390/biomedicines12061234 (PMC11201257; doi:10.3390/biomedicines12061234)
Supplement: Supplementary file 1 [file biomedicines-12-01234-s001.zip › biomedicines-2891800-supplementary.pdf]

Supplementary material

Table S1. Frequency and persistence of anemia in the study population

Baseline anemia	19	Cured in FU (baseline anemia but no FU anemia)	8/19
		Persistent in FU ‡	7/19
		Unknown (follow-up information missing)Ł	4/19
FU anemia	11	New onset anemia (no anemia at baseline)	3/11
		Persistent in FU ‡	7/11
		Unknown (baseline information missing)Ł	1/11

‡ a computed variable; baseline anemia =1 (Yes), if FU anemia = 1 (Yes)

Ł either baseline anemia data or FU anemia data were missing

Table S2. Characteristics of study population according to hemoglobin level change

Population characteristics		Unknown Hb change status (N= 15)	Clinically notable decresead Hb (N=8)	Clinically notable increased Hb or unchanged Hb (N=72)
Demographic characteristics				
Age (mean, SD)		52.07 (7.23)	56.75 (5.52)	54.29
Male (N, %)		6 (40.0%)	4 (50.00%)	38 (52.8%)
Ethnicity (N, %)	Afiican	1 (6.7%)	0 (0.00%)	7 (9.7%)
	Asian	0 (0.00%)	0 (0.00%)	3 (4.2%)
	Caucasian	12 (80.0%)	4 (50.00 %)	50 (69.4%)
	Latin-american	0 (0.00%)	2 (25.00%)	1 (1.4%)
	Others	1 (6.7%)	0 (0.0%)	5 (6.9%)
	Unknown	1 (6.7%)	2 (25.00%)	6 (8.3%)
Clinical characteristics				
Suffering from comorbidities (N, %)		83 (89.2%)	7 (87.5%)	64 (88.9%)
BMI (mean, SD)		30.13 (6.68)	30.84 (3.37)	30.54 (5.20)
Dominant SARS-CoV-2 variant (N, %)	Alpha	7 (46.7%)	3 (37.5%)	33 (45.8%)
	Delta	7 (46.7%)	3 (37.5%)	31 (43.1%)
	Omicron	1 (6.7%)	2 (25.0%)	8 (11.1%)
Hospitalized (N, %)		5 (33.3%)	8 (100.0%)	71 (100.0%)
Hospital stay (days) (median, percentile 25 - 75)		0 (0.00 - 6.00)	17 (4.50-31.75)	8.00 (6.00 – 16.00)
ICU admission (N, %)		0 (0.0%)	3 (37.5%)	24 (33.8%)
ICU stay (days) (median, percentile 25 - 75)		0 .0 (0.0 - 0.0)	0 (0 - 19.25)	0.0 (0.0 - 5.0)
WHO severity index (N, %)	Mild	10 (66.7%)	0 (0.0%)	0 (0.0%)
	Moderate	5 (33.3%)	5 (62.5%)	47 (65.3.4%)
	Severe	0 (0.0%)	3 (37.5%)	23 (31.9%)
	NA	0 (0.0%)	0 (0.0%)	2 (2.8%)
Average FSS during the 3-6 months visit (median, percentile 25 - 75)		6.44 (3.67 – 6.78)	5.44 (4.11 – 6.00)	5.44 (4.06 - 6.17)
Suffering from fatigue (Average FSS ≥ 4) during the 3-6 months visit (N, %)		11 (73.3%)	6 (75.0%)	64 (88.9%)

Table S3. Correlations between hemoglobin levels and demographic and clinical characteristics.

Variable	Mean/Median (SD/Percentile 25-75)	cc with Hb change	N*	cc with baseline Hb	N	cc with FU Hb	N
Age (mean, SD)	54.15 (6.18)	0.009	59	0.087	84	0.136	68
ICU stay duration (median, percentile 25 - 75)	8.00 (4.00 - 15.25)	0.341	58	-0.077	83	0.259	67
Hospital stay duration (median, percentile 25 - 75)	0.00 (0.00 - 4.00)	0.268	58	-0.039	83	0.214	67
BMI(mean, SD)	30.50 (5.28)	-0.193	58	0.050	83	-0.092	67
*number of cases that correlation analysis between them and Hb change has been performed.							

Table S4. Results of logistic regression model for Hemoglobin and fatigue (average FSS ≥ 4) for cases with GFR of mild renal dysfunction or better (≥60 mL/min/1.73m2, N=56)

	Unadjusted	Adjusted*
	OR‡ (95% CI)	OR (95% CI)
Baseline Hb levels	1.050 (0.518 - 2.127)	0.974 (0.448 - 2.116)
Follow-up Hb levels	0.544 (0.205 - 1.448)	0.475 (0.159 - 1.419)
Hb change	0.478 (0.155 - 1.473)	0.467 (0.150 - 1.452)
Baseline anemia	1.867 (0.200 - 17.459)	1.776 (0.181 - 17.453)
FU anemia	1.200 (0.124 - 11.583)	1.225 (0-123 - 12.252)
*Adjusted for age and sex<br>‡ Per 1 mmol/L increase in Hb levels		

Table S5. Results of logistic regression model for Hemoglobin and fatigue (average FSS ≥ 4) after excluding the case with extreme outlier for hemoglobin

	Unadjusted	Adjusted*
	OR‡ (95% CI)	OR (95% CI)
Baseline Hb levels	0.693 (0.387 - 1.243)	0.800 (0.432 - 1.481)
Follow-up Hb levels	0.406 (0.154 - 1.071)	0.374 (0.131 - 1.069)
Hb change	0.389 (0.137 - 1.104)	0.389 (0.135 - 1.124)
Baseline anemia	1.116 (0.314 - 3.970)	1.433 (0.383 – 5.365)
FU anemia	2.118 (0.234 - 19.205)	2.251 (0.244 - 20.746)
*Adjusted for age and sex<br>‡ Per 1 mmol/L increase in Hb levels		

Table S6. Frequency of SARS-CoV-2 vaccination (main dosage) in the study population

			First dosage	Second dosage
SARS-CoV-2 vaccination (N=95)	Yes	Before infection	27 (28.4%)	15 (15.8%)
		After infection	38 (40.0%)	25 (26.3%)
		Unknown/NA	30 (31.6%)	55 (57.9%)
		Total	66 (69.5%)	41 (43.2%)
	No	Total	29 (30.5%)	54 (56.8%)

Table S7. Type of SARS-CoV-2 vaccination in the study population

Vaccination type	AstraZeneca	BioNTech/Pfizer (Comirnaty)	Janssen	Moderna	Unknown/others	NA
Frequency (n,%)	10 (10.5%)	48 (50.5%)	4 (4.2%)	5 (5.3%)	11(11.6%)	17 (17.9%)
